# Efficacy of Hangeshashinto in the Prevention of Chemotherapy-Induced Diarrhea: A Systematic Review and Meta-Analysis

**DOI:** 10.7759/cureus.50377

**Published:** 2023-12-12

**Authors:** Takeru Takahashi, Koshi Nagai, Kazumasa Kotake

**Affiliations:** 1 Pharmacy Department, Akita City Hospital, Akita, JPN; 2 Pharmacy Department, Tokyo Metropolitan Police Hospital, Tokyo, JPN; 3 Pharmacy Department, Okayama Saiseikai General Hospital, Okayama, JPN

**Keywords:** chemotherapy-induced diarrhea, prevention, cancer, chemotherapy, diarrhea, hangeshashinto

## Abstract

Hangeshashinto has attracted attention owing to its potential to prevent chemotherapy-induced diarrhea. However, studies on the efficacy of Hangeshashinto have had conflicting results. Evaluating the efficacy of Hangeshashinto may contribute to reducing the use and adverse events caused by drug therapy for chemotherapy-induced diarrhea. Medical Literature Analysis and Retrieval System Online (MEDLINE), PubMed, Ichushi, the Cochrane Central Register of Controlled Trials (CENTRAL), and ClinicalTrials.gov were searched to retrieve all the relevant studies. Randomized controlled trials (RCTs) comparing the administration of Hangeshashinto with that of other treatments in patients with cancer receiving chemotherapy were included. The primary outcome was severe (grade 3-4) diarrhea assessed using the Common Terminology Criteria for Adverse Events. The secondary outcome was mild (grade 0-2) diarrhea. Out of 324 records identified, three studies were selected for the meta-analysis. Irinotecan was used for chemotherapy in all these studies. Hangeshashinto did not reduce the incidence of severe diarrhea compared with other treatments (risk ratio (RR) 0.40, 95% confidence interval (CI) 0.11-1.41, P = 0.15; low-quality evidence). Moreover, Hangeshashinto did not reduce the incidence of mild diarrhea (RR 1.35, 95% CI 0.87-2.09, P = 0.18; low-quality evidence). However, in the subgroup analysis compared with no treatment, the Hangeshashinto group had a significantly lower incidence of severe diarrhea (RR 0.17, 95% CI 0.03-0.88, P = 0.03; low-quality evidence). At present, insufficient evidence exists to support the claim that Hangeshashinto prevents diarrhea caused by irinotecan-based chemotherapy.

## Introduction and background

Diarrhea is a major side effect of chemotherapy and occurs in 50%-80% of patients receiving chemotherapy. More than 30% of patients receiving irinotecan or 5-fluorouracil experience severe diarrhea [1]. Severe diarrhea can lower the quality of life, increase anxiety, and interrupt treatment [2]. Therefore, treatment and prevention of severe diarrhea caused by irinotecan and 5-fluorouracil are essential.

Several drugs are used to treat chemotherapy-induced diarrhea, including loperamide, atropine, and octreotide [3, 4]. However, aggressive use of these drugs may lead to adverse events such as ileus and QT prolongation [3, 5]. Therefore, preventing the onset of severe chemotherapy-induced diarrhea is important. The administration of probiotics or activated charcoal has been reported to prevent severe diarrhea; however, the efficacy of these drugs is unclear [3, 6].

Hangeshashinto is a traditional Japanese herbal medicine used for diarrhea, anorexia, nausea, and vomiting [7-12]. This herbal medicine may cause pseudo-hyperaldosteronism and skin rash, but their frequency is low, and serious adverse events are extremely rare [13]. In addition, Hangeshashinto is less expensive and could be a potential alternative to existing therapeutic agents for chemotherapy-induced diarrhea [14-16]. However, studies on the efficacy of Hangeshashinto in preventing chemotherapy-induced diarrhea have reported conflicting results [17]. Evaluating the effects of Hangeshashinto may reduce the use of and adverse events caused by drug therapy for chemotherapy-induced diarrhea. Therefore, we performed a systematic review and meta-analysis to investigate whether Hangeshashinto prevents diarrhea in patients with cancer receiving chemotherapy.

## Review

Methods

Search Methods for Identification of Studies

This systematic review and meta-analysis was conducted according to the Preferred Reporting Items for Systematic Reviews and Meta-Analysis (PRISMA) protocol [18].

Only randomized controlled trials (RCTs) were included. The study population included patients with cancer who were undergoing chemotherapy. Patients who received Hangeshashinto were considered the intervention group, and those receiving no medication or other drugs were considered comparators. There were no restrictions on the language or publication status. Databases, including Medical Literature Analysis and Retrieval System Online (MEDLINE) via PubMed, Ichushi, the Cochrane Central Register of Controlled Trials (CENTRAL), and ClinicalTrials.gov, were searched comprehensively to retrieve all relevant studies published until October 11, 2022. Additionally, the reference lists of clinical guidelines were searched for relevant studies. Moreover, we contacted the authors of the trials that had not reported all the outcomes to obtain unpublished data. The search strategy for the databases is shown in Table 1.

**Table 1:** Search strategies for databases CENTRAL: Cochrane Central Register of Controlled Trials

Database	Search string
PubMed	(Neoplasms［Mesh Terms］） OR （Neoplas＊［Title/Abstract]) OR (Tumor[Title/Abstract]) OR (Tumour*[Title/Abstract]) OR (cancer*[Title/Abstract]) OR (Malignanc*[Title/Abstract]) OR (Malignant*[Title/Abstract])) OR ((Leukemia[Mesh Terms]) OR (Leukemias[Title/Abstract]) OR (Leucocythaemia[Title/Abstract]) OR (Leucocythemia[Title/Abstract])) AND ((Hangeshashinto[Title/Abstract]) OR (TJ-14[Title/Abstract]) OR (Banxia Xiexin[Title/Abstract]) OR (Banxiaxiexin[Title/Abstract]) OR (Ban Xia Xie Xin[Title/Abstract]) OR (Banha-Sasim-Tang[Title/Abstract])) AND ((randomized controlled trial[Publication Type]) OR (controlled clinical trial[Publication Type]) OR (randomized[Title/Abstract]) OR (placebo[Title/Abstract]) OR (drug therapy[MeSH Subheading]) OR (randomly[Title/Abstract]) OR (trial[Title/Abstract]) OR (groups[Title/Abstract])) NOT ((animals[Mesh Terms]) NOT (humans[Mesh Terms]))
Ichushi	(([Neoplasms]/Thesaurus) OR ([Leukemia]/Thesaurus)) AND ((Hangeshashinto /Thesaurus) OR (Hangeshashinto /All Field)) in Japanese.
CENTRAL	(“Hangeshashinto” OR “TJ-14” OR “Banxia Xiexin” OR “Ban Xia Xie Xin” OR “Banha-Sasim-Tang” OR “Pinellia Heartdraining” OR “Pinellia Decoction for Draining the Heart” [Title/Abstract/Keyword])
ClinicalTrials.gov	(Other terms: Hangeshashinto OR TJ-14 OR Banxia Xiexin OR Ban Xia Xie Xin OR Banha-Sasim-Tang OR Pinellia Heartdraining OR Pinellia Decoction for Draining the Heart)

All titles and abstracts were screened according to the inclusion criteria. Full-text reviews were performed as needed. Outcome measurements, data extraction, quality assessment, data integration, and statistical analyses were performed independently by two researchers (TT and KN), and any discrepancies were discussed with another researcher (KK).

The study protocol was registered at the University Hospital Medical Information Network (UMIN) Clinical Trials Registry (UMIN 000049104).

Outcome Measures

The primary outcome was severe (grade 3-4) diarrhea assessed using the Common Terminology Criteria for Adverse Events (CTCAE). The secondary outcome was mild (grade 0-2) diarrhea. If the comparators for Hangeshashinto differed between studies, a subgroup analysis was performed for each comparator.

Data Extraction and Quality Assessment

We collected the following data: authors, date of online publication, country, study design, population size, number of dropouts, patient characteristics (sex and age), cancer type, Eastern Cooperative Oncology Group (ECOG) performance status (PS), outcomes, details of interventions, dose of Hangeshashinto, and chemotherapy regimens.

We assessed the risk of bias in the included studies using the Cochrane tool for assessing the risk of bias [19]. We categorized each potential domain of bias as high, low, or unclear and summarized each domain’s bias risk determination in a “risk of bias” table. The Grading of Recommendations Assessment, Development, and Evaluation (GRADE) approach was used to assess the quality of evidence for each outcome [20]. The certainty of the evidence was classified as “very low,” “low,” “moderate,” or “high” using the GRADE criteria.

Data Synthesis and Statistical Analysis

Meta-analysis was performed with a random-effects model using risk ratios (RR) with 95% confidence intervals (CI). Heterogeneity was assessed visually with a forest plot and statistically with a Chi-squared test. Heterogeneity was quantified using the I2 statistic and interpreted as follows: 0%-40%: low heterogeneity; 30%-60%: moderate heterogeneity; 50%-90%: substantial heterogeneity; and 75%-100%: considerable heterogeneity. Publication bias was assessed by visual inspection of funnel plots or Egger’s test [21].

All statistical analyses were performed using the Review Manager (RevMan) software 5.4.1 (The Cochrane Collaboration, London, UK). Statistical significance was set at P <0.05.

Results

Selection of Studies

**Figure 1:**
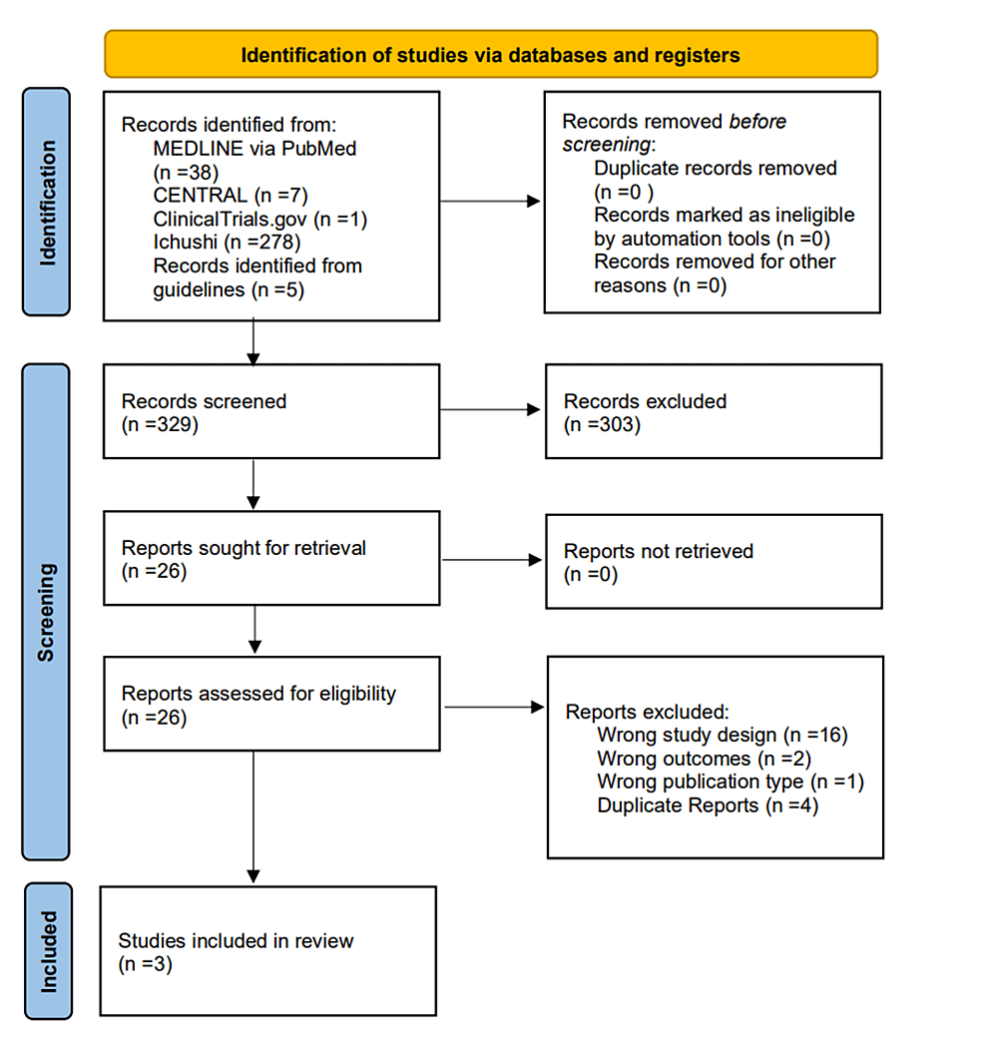
A PRISMA flow diagram showcasing the process of study selection CENTRAL: Cochrane Controlled Register of Trials; MEDLINE: Medical Literature Analysis and Retrieval System Online; PRISMA: Preferred Reporting Items for Systematic Reviews and Meta-Analyses

Figure 1 shows the Preferred Reporting Items for Systematic Reviews and Meta-Analyses (PRISMA) flow diagram of the patient selection process.

The search identified a total of 324 records. Five records were identified from the guidelines. Of those, 303 records were excluded because they did not fit our inclusion criteria for the title and abstract review. The remaining 26 reports were screened by full-text review. Finally, three studies were selected for meta-analysis [16,17,22].

Characteristics of the Included Studies

Table 2 presents the characteristics of the included studies.

**Table 2:** Characteristics of studies included in the meta-analysis ECOG: Eastern Cooperative Oncology Group; CDDP: cis-diamminedichloroplatinum(II); CPT-11: irinotecan; S-1: an oral anticancer drug that combines tegafur, gimeracil, and potassium oteracil; FOLFIRI: CPT-11 in combination with 5-fluorouracil and leucovorin; oral alkalization; sodium bicarbonate: 1.8 g/day; and ursodeoxycholic acid: 300 mg/day.

Author (Year)	Study design (Country)	Hangeshashinto dose	Control	Cancer	Chemotherapy	Total number of patients (Mean age)	Proportion of men	ECOG performance status (N)
Mori K et al. (2003)[16]	Open-label (Japan)	7.5g/day	No medication	Lung	CDDP/CPT-11	41 (61 years)	76%	0 (5), 1 (30), 2 (6)
Hibi S et al. (2009)[22]	Open-label (Japan)	7.5g/day	No medication	Colorectal Gastric	S-1/ CPT-11	20 (69 years)	60%	0 (13), 1 (7)
Yamazaki K et al.(2019)[17]	Open-label (Japan)	7.5g/day	Oral alkalization	Colorectal	FOLFIRI	29 (57 years)	55%	0 (27), 1 (2)

All the included studies were published between 2003 and 2019. Two studies were published in English and one in Japanese [22]. These studies used irinotecan in chemotherapy. The cancer types and chemotherapy regimens of the patient populations differed between the studies. All patients had an ECOG PS of 0-2. The dose of Hangeshashinto was 7.5 g/day in all studies; however, the duration of administration differed among the studies. All studies were open-label and conducted in Japan. Mori et al. used CTCAE version 2, and CTCAE version 3 was used in other previous studies.

Quality Assessment

**Figure 2:**
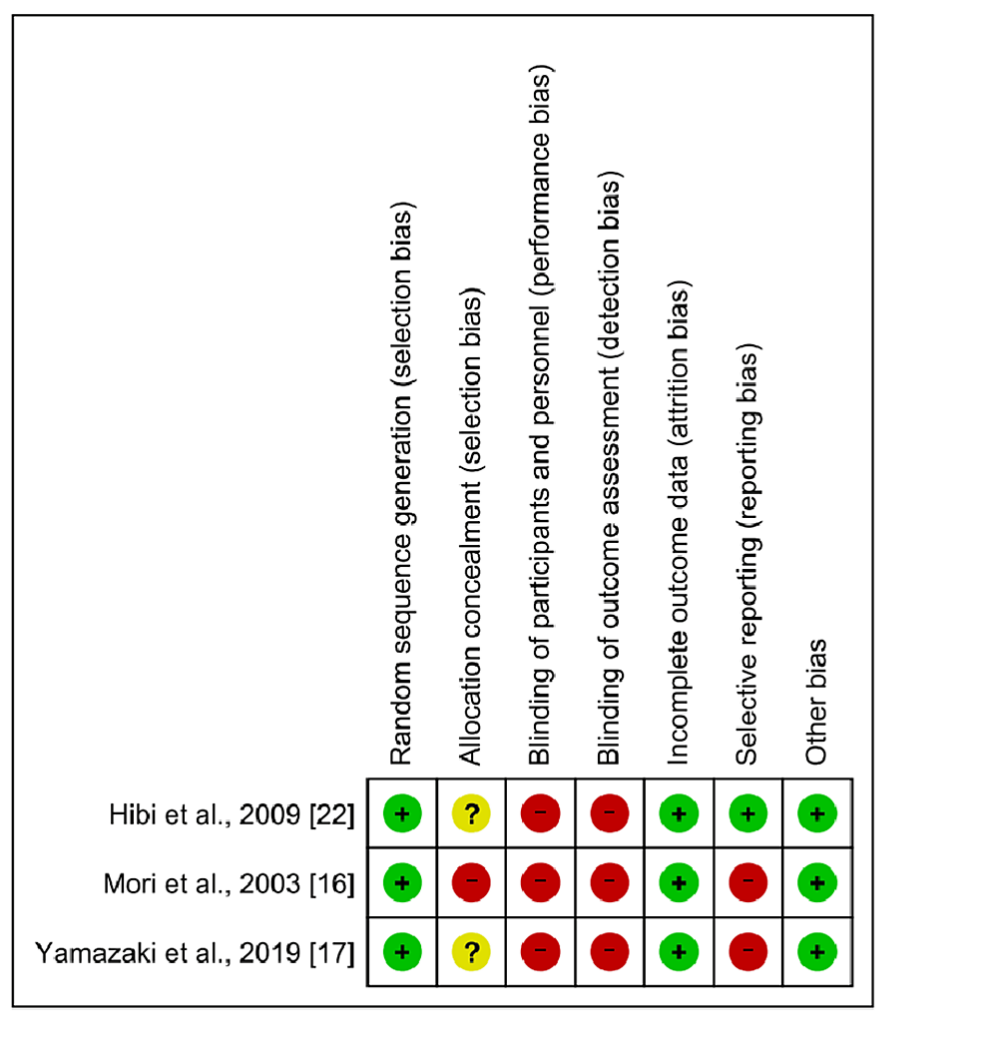
Risk of bias summary

The overall risk of bias was considered high (Figure 2).

The details of the randomization methodology were “unclear” in two studies. In addition, all studies were rated “high” for performance bias and detection bias because of their open-label design. Due to the limited reporting of outcome data, two studies were rated "high" for reporting bias. Publication bias could not be assessed due to the small number of included studies.

Primary Outcome: Effect of Hangeshashinto on Grade 3-4 Diarrhea

**Figure 3:**
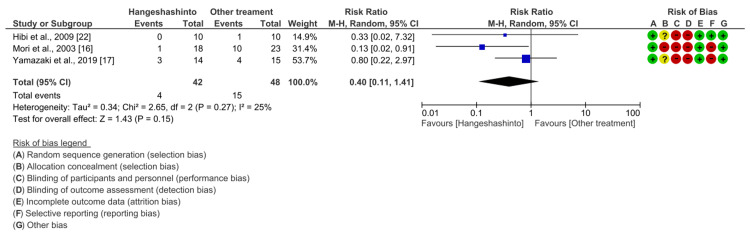
Forest plot comparing the incidence of grade 3-4 diarrhea between Hangeshashinto and other treatments

Data on the occurrence of grade 3-4 diarrhea in patients receiving chemotherapy were available for all three studies. Compared with other treatments, Hangeshashinto did not significantly reduce the incidence of grade 3-4 diarrhea (RR 0.40, 95% CI 0.11-1.41, P = 0.15). Heterogeneity was low (I2 = 25%) (Figure 3).

Secondary Outcome: Effect of Hangeshashinto on Grade 0-2 Diarrhea

**Figure 4:**
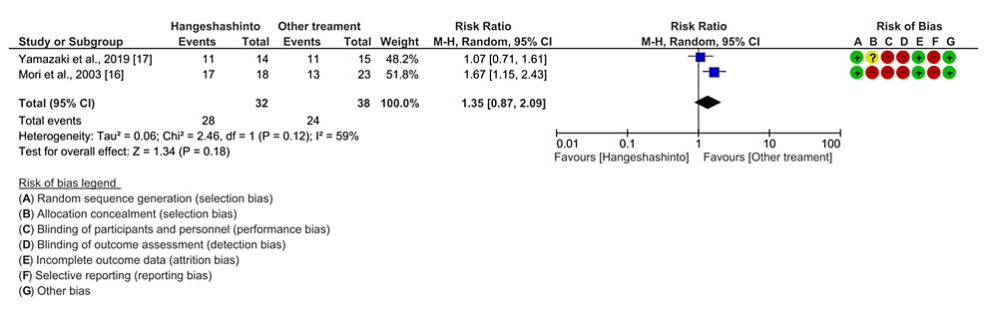
Forest plot comparing the incidence of grade 0-2 diarrhea between Hangeshashinto and other treatments

Two studies involving 70 patients were included in the analyses. Compared with other treatments, Hangeshashinto did not significantly reduce the incidence of grade 0-2 diarrhea (RR 1.35, 95% CI 0.87-2.09, P = 0.18). Heterogeneity was moderate (I2 = 59%) (Figure 4).

Subgroup Analysis: Effect of Hangeshashinto on Grade 3-4 Diarrhea Compared with No Treatment

**Figure 5:**
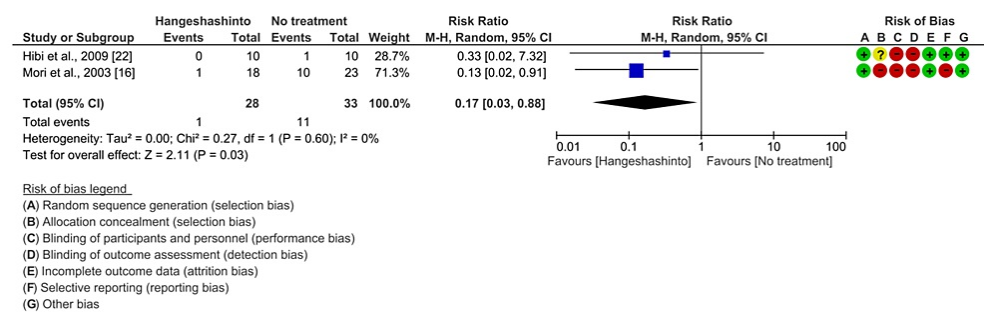
Forest plot of subgroup analysis comparing the incidence of grade 3-4 diarrhea between Hangeshashinto and no treatment

In the subgroup analysis, 61 patients from two studies were included in the meta-analysis after excluding studies in which alkalinizing agents were administered to the control group. The Hangeshashinto group had a significantly lower incidence of grade 3-4 diarrhea than the no-treatment group (RR 0.17, 95% CI 0.03-0.88, P = 0.03). Heterogeneity was low (I2 = 0%) (Figure 5).

Certainty Measure Using the GRADE Approach

Table 3 presents the GRADE evidence profile for the outcomes of this review.

**Table 3:** GRADE evidence profile and summary of findings The risk (and its 95% confidence interval) for the intervention group is based on the assumed risk in the comparison group and the relative effect of the intervention (and its 95% confidence interval). CI: confidence interval; GRADE: Grading of Recommendations Assessment, Development, and Evaluation; RCT: randomized controlled trial; RR: risk ratio. The GRADE Working Group grades of evidence: High certainty: We are very confident that the true effect lies close to that of the estimate of the effect; Moderate certainty: We are moderately confident in the effect estimate. The true effect is likely to be close to the estimate of the effect, but there is a possibility that it is substantially different; Low certainty: Our confidence in the effect estimate is limited. The true effect may be substantially different from the estimate of the effect; Very low certainty: We have very little confidence in the effect estimate. The true effect is likely to be substantially different from the estimate of the effect. ^a ^Downgraded by two levels because of a very small sample size and a lack of blinding.

Outcome	Participants (number and study design)	Certainty	I^2^ index	Summary of findings		
				Relative effect (95% CI)	Risk with control*	Risk difference* (95% CI)
Grade 3-4 diarrhea	90 (3 RCTs)	⨁⨁⊖⊖ Low^a^	25%	RR 0.40 (0.11–1.41)	313	–188 (–278 to 128)
Grade 0-2 diarrhea	70 (2 RCTs)	⨁⨁⊖⊖ Low^a^	59%	RR 1.35 (0.87–2.09)	632	221 (–82 to 688)
Subgroup analysis of grade 3-4 diarrhea	61 (2 RCTs)	⨁⨁⊖⊖ Low^a^	0%	RR 0.17 (0.03–0.88)	333	–277 (–323 to –40)

Prevention of grade 3-4 diarrhea with Hangeshashinto in patients with cancer receiving chemotherapy and its subgroup analysis and prevention of grade 0-2 diarrhea were downgraded by two levels of certainty due to the small sample size and lack of blinding. These outcomes were rated as "low quality" in evidence because of the serious risk of bias.

Discussion

Our study suggests that Hangeshashinto administration does not reduce the incidence of chemotherapy-induced diarrhea. However, the subgroup analysis comparing Hangeshashinto administration with no treatment suggests that Hangeshashinto might reduce the incidence of severe diarrhea.

An observational study reported that Hangeshashinto administration might be effective against chemotherapy-induced diarrhea [7]. However, previously reported RCTs examining the efficacy of Hangeshashinto for chemotherapy-induced severe diarrhea were contradictory, with some reporting that it prevented severe diarrhea and others saying that it did not compare with no treatment [16, 17, 22]. Thus, it was unclear whether Hangeshashinto administration could prevent chemotherapy-induced severe diarrhea.

As a primary outcome, Hangeshashinto administration, compared with other treatments, did not prevent chemotherapy-induced severe diarrhea. This result is inconsistent with that of previous studies showing that Hangeshashinto administration may prevent severe diarrhea caused by irinotecan [7]. Moreover, irinotecan was used in all three included studies, but only one study, reported by Mori et al., significantly prevented severe diarrhea. Hibi et al. reported that severe diarrhea occurred only in the non-treatment group and only in one patient. There was no difference in the incidence of severe diarrhea reported by Yamazaki et al. compared to oral alkalization. This indicates that there is limited evidence regarding the efficacy of Hangeshashinto for severe diarrhea with irinotecan-based chemotherapy.

As a secondary outcome, we found that Hangeshashinto administration did not prevent mild diarrhea caused by chemotherapy. There are two reasons for this finding. First, the study by Yamazaki et al. used oral alkalinization as a comparator. Oral alkalization may decrease the incidence of severe diarrhea [23]. Therefore, there may have been no difference in the frequency of diarrhea between the oral alkalization and Hangeshashinto groups. Second, in the study by Mori et al., Hangeshashinto was found to be highly effective against severe diarrhea, which may have increased the proportion of patients with relatively mild diarrhea.

Subgroup analysis showed that Hangeshashinto administration effectively reduced chemotherapy-induced severe diarrhea as compared with no treatment. However, this finding was strongly influenced by the results of the study by Mori et al. and should be considered carefully. Mori et al. showed that Hangeshashinto had a large effect on severe diarrhea caused by cancer chemotherapy. This may be related to the duration and timing of the Hangeshashinto administration. Hangeshashinto was administered three days prior to the start of chemotherapy and continued for at least 21 days. In contrast, Hibi et al. reported that Hangeshashinto was administered for only three days from the start of chemotherapy. Previous studies have shown that Hangeshashinto administered for a week or longer is effective in reducing diarrhea frequency [14]. In addition, starting Hangeshashinto two to three days before irinotecan administration has been shown to reduce diarrhea frequency [7]. Therefore, it may be important to continue the administration of Hangeshashinto before the administration of irinotecan. Diarrhea caused by irinotecan is a serious problem that can lower the quality of life and interrupt treatment in patients with cancer [2]. Therefore, Hangeshashinto can be a potential treatment strategy for irinotecan-induced severe diarrhea, as compared with no treatment, given the duration and timing of administration.

This study has several limitations. First, all studies had very small sample sizes, indicating that the obtained results were unreliable. Moreover, the risk of bias and imprecision was severe, and the outcomes obtained were judged to be low-quality evidence. This indicates the need for high-quality, large-sample-size RCTs to be conducted. Second, only irinotecan chemotherapy was administered in the included studies. Thus, the results of this review are not applicable to all types of malignancies or chemotherapies. To our knowledge, this is the first review to evaluate the efficacy and safety of Hangeshashinto for treating severe diarrhea in patients undergoing chemotherapy.

## Conclusions

At present, insufficient evidence exists to support the claim that Hangeshashinto prevents diarrhea caused by irinotecan-based chemotherapy. However, Hangeshashinto is safe and inexpensive. Therefore, further RCTs might be beneficial in medical economics and potentially become a treatment strategy for chemotherapy-induced diarrhea.
